# Pro-Inflammatory Stimuli Influence Expression of Intercellular Adhesion Molecule 1 in Human Anulus Fibrosus Cells through FAK/ERK/GSK3 and PKCδ Signaling Pathways

**DOI:** 10.3390/ijms20010077

**Published:** 2018-12-25

**Authors:** Bor-Ren Huang, Da-Tian Bau, Tzu-Sheng Chen, I-Chen Chuang, Cheng-Fang Tsai, Pei-Chun Chang, Horng-Chaung Hsu, Dah-Yuu Lu

**Affiliations:** 1 Graduate Institute of Clinical Medical Science, China Medical University, Taichung 40402, Taiwan; smallblue525@hotmail.com (B.-R.H.); dtbau@mail.cmu.edu.tw (D.-T.B.); 2 Neurosurgery Department, Taichung Tzu Chi Hospital, Buddhist Tzu Chi Medical Foundation, Taichung 42743, Taiwan; 3 School of Medicine, Tzu Chi University, Hualien 97002, Taiwan; 4 Department of Pathology, Taichung Tzu Chi Hospital, Buddhist Tzu Chi Medical Foundation, Taichung 42743, Taiwan; 4sheng@gmail.com; 5 Department of Pharmacology, School of Medicine, China Medical University, Taichung 40402, Taiwan; pansy7799@msn.com; 6 Department of Biotechnology, Asia University, Taichung 41354, Taiwan; tsaicf@asia.edu.tw; 7 Department of Bioinformatics, Asia University, Taichung 41354, Taiwan; pcchang@asia.edu.tw; 8 Department of Orthopedic Surgery, China Medical University Hospital, Taichung 40402, Taiwan; d4749@mail.cmuh.org.tw; 9 Department of Photonics and Communication Engineering, Asia University, Taichung 41354, Taiwan

**Keywords:** intervertebral disc, degeneration, anulus fibrosus, CCL2, ICAM1

## Abstract

Objective: Intervertebral disc (IVD) degeneration and disc herniation are major causes of lower back pain, which involve the presence of inflammatory mediators and tissue invasion by immune cells. Intercellular adhesion molecule 1 (ICAM1, also termed CD54) is an adhesion molecule that mediates cell-cell interactions, particularly between immune cells and target tissue. The aim of this study was to examine the intracellular signaling pathways involved in inflammatory stimuli-induced ICAM1 expression in human anulus fibrosus (AF) cells. Methods: Quantitative reverse transcription-polymerase chain reaction (qPCR), western blotting, and flow cytometry were performed to dissect the roles of different signaling pathways in inflammatory stimuli-mediated ICAM1 expression. Results: Using qPCR and western blot analyses, a significant increase in ICAM1 expression was observed in AF cells after stimulation of lipopolysaccharide (LPS) plus interferon-gamma (IFNγ) in a time-dependent manner. Flow cytometry revealed ICAM1 upregulation on the surface of AF cells. Importantly, LPS plus IFNγ treatment also significantly promoted Chemokine ligand (CCL)2 expression, but not CCL3. The enhanced ICAM1 expression was abolished after incubation with antibody against CCL2. In AF cells, treatment with LPS plus IFNγ activated the FAK/ERK/GSK3 signaling pathways, promoted a time-dependent increase in PKCδ phosphorylation, and promoted PKCδ translocation to the nucleus. Treatment with the pharmacological PKCδ inhibitor; rottlerin, effectively blocked the enhanced productions of ICAM1 and CCL2. Conclusions: Inflammatory stimuli in AF cells are part of a specific pathophysiology in IVD degeneration and disc herniation that modulates CCL2/ICAM1 activation through the FAK/ERK/GSK3 and PKCδ signaling pathways in AF cells.

## 1. Introduction

Intervertebral discs (IVDs) have sparse nerve endings and blood vessels consisting of a central gelatinous proteoglycan-rich nucleus pulposus (NP) and an outer multi-lamellar collagen-rich fibrocartilage annulus fibrosus (AF), which is connected by the cartilaginous endplates of the vertebral bodies [1]. Cells in the NP area are exposed to highly hydrostatic pressures, and cells within the AF are resisted to large tensile strain [2]. In the healthy IVDs, the NP exerts a hydrostatic pressure against the constraining AF, which allows the disc into twisting and bending motions. However, degenerative changes in the biomechanical and structural properties of NP and AF in humans usually occur with aging, resulting in the loss of integrity and interplay of IVD and serious degenerative diseases like degenerative disc disease and low back pain. The etiology of low back pain as a symptom of degenerative disc disease (DDD) is complex and appears to be a combination of mechanical deformation and the presence of an inflammatory response [3]. Moreover, IVD degeneration can precede disc herniation, which results in an inflammatory response and further pain [4]. Disc herniation is a major cause of low back pain that may be caused by the mechanical compression of the lumbar nerve root, as well as the local inflammation of discs [5,6]. The initial phase of disc degeneration is characterized by an overall shift that compromises the structural integrity of the tissue, which leads to an inflammatory response characterized by the invasion of immune cells into tissues [7]. Several cytokines have been considered to be as the pathogenesis of IVD degeneration and herniation, such as IL-1 and TNFα [8,9]. Furthermore, a previous report suggests that inflammatory response that occurs around hernia tissue plays a critical role in herniated disc regression [10]. However, the nucleus cell types in rodent animals differ from those of adult humans, which are not good models to fully elucidate human degeneration. Therefore, a greater understanding of regulatory mediators involved in inflammatory responses of IVDs is required prior to the development of successful therapeutic approaches to disc herniation. 

Intercellular adhesion molecule 1 (ICAM1), inducible surface glycoprotein regulates the interactions of adhesion, migration, and invasion of neutrophils and/or lymphocytes during tissue injury that causes immune responses and subsequent damage [11,12]. Previous studies indicated that ICAM1 expression may be important to the adhesive functions of immune cells as well as for the recruitment of additional cell types, such as monocytes, to the site of degeneration. Early studies revealed macrophage and lymphocyte infiltration with pro-inflammatory cytokine expression in pathologic IVD tissues [7]. Recently, a bioinformatics analysis of gene expression profiles of degenerative IVD cells revealed that ICAM1 may play a role in the development of disc herniation [13]. Importantly, ICAM1 has also been found in herniated intervertebral discs of lumbar spine tissues [14]. These results suggest that the ICAM1 adhesion molecule may play a critical role in the attraction of immune cells in degenerated IVDs.

Chemokines can be stimulated by pro-inflammatory cytokines or pathogenic stimuli arising in inflammatory tissues, which trigger immune cell infiltration and consequently influence chemotaxis, cell proliferation, and several biological activities [15]. Chemokine ligand (CCL)2/monocyte chemoattractant protein 1 (MCP-1), a ligand of chemokine receptor (CCR)2, belongs to the CC chemokine family. High levels of CCL2 contribute to the infiltration of monocytes and their differentiation into macrophages with tissue remodeling functions in the epidural compartment after intervertebral disc extrusion [16]. Importantly, CCL2/CCR2 signaling in a rat model of lumbar disc herniation may contribute to neuropathic pain through the regulation of macrophage infiltration [17]. CCL3, which is also known as macrophage inflammatory protein 1-alpha (MIP-1-α) in NP, has been reported to promote macrophage infiltration disc degeneration [18]. A recent study reported that IVD degeneration-associated macrophages induced molecular changes via upregulation of CCL2 and CCL3 in NP cells [19]. Interestingly, the expression of CCL3 in IVD degeneration in a canine model was significantly correlated with pain severity [16]. The regulatory mechanism of these chemokines during IVD degeneration and disc herniation is unclear and there is currently no specific treatment for DDD [20]. 

The study aimed to investigate the relationship between chemokines, expression of ICAM1 in human anulus fibrosus (AF) cells, and inflammation-associated IVD degeneration, and to investigate the underlying molecular mechanisms. The results showed that pro-inflammatory stimuli induced ICAM1 expression in AF cells and promoted CCL2 expression. The enhancement of CCL2-dependent ICAM1 expression occurs through the JAK1/FAK/ERK/GSK3 and PKCδ signaling pathways in AF cells, which may contribute to immune cell recruitment and disc degenerative diseases. 

## 2. Results

### 2.1. Regulatory Effect of CCL2 on ICAM1 Expression in IVD Cells

**Figure 1:**
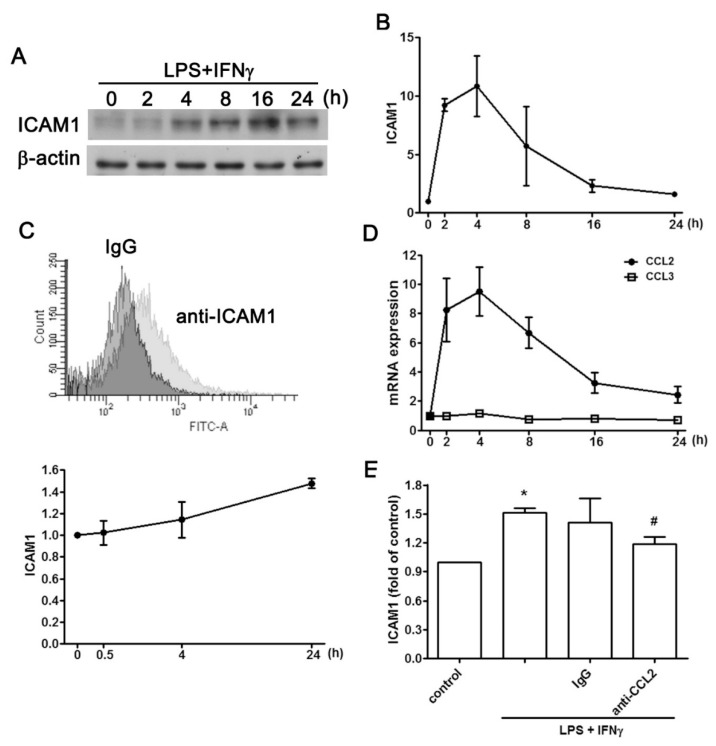
CCL2 expression is involved in ICAM1 upregulation in AF cells. Stimulation with LPS plus IFNγ increased total and cell surface expression of ICAM1 as determined by western blotting (**A**), and flow cytometry (**C**). (**B**) AF cells were stimulated with LPS plus IFNγ for indicated times, and the mRNA level of *ICAM1* was determined by real-time PCR. (**D**) Following stimulation of AF cells with LPS plus IFNγ, the mRNA levels of *CCL2* and *CCL3* were determined by real-time PCR. (**E**) After incubation with CCL2-neutralizing antibody or control IgG followed by stimulation with LPS plus IFNγ, ICAM1 cell surface expression was determined by flow cytometry. Note that CCL2 expression was increased in stimulated AF cells, and that CCL2 was an endogenous regulatory mediator in ICAM1 expression. The results are expressed as the mean ± standard error of the mean from three independent experiments. * *p* < 0.05 compared with the control group. # *p* < 0.05 compared with the LPS/ IFNγ treatment group.

Human degenerated IVD samples displayed enhanced ICAM1 intensity. Inflammatory stimuli of AF cells in the form of LPS plus IFNγ, markedly elevated ICAM1 protein expression in a time-dependent manner in western blots (Figure 1A), and flow cytometry (Figure 1C). mRNA expression of *ICAM1* increased after a 4-h stimulation with LPS plus IFNγ, and it gradually decreased thereafter (Figure 1B). The expression of *CCL2* and *CCL3* pro-inflammatory chemokines was also measured. *CCL2* expression increased after a 4-h exposure to LPS plus IFNγ, while the expression of *CCL3* was not affected (Figure 1D). To investigate the regulation between CCL2 and ICAM1, a neutralizing antibody against CCL2 was used to evaluate the expression of ICAM1. The CCL2 neutralizing antibody abrogated the protein expression of cell surface ICAM1 induced by LPS plus IFNγ (Figure 1E). These findings suggested that LPS plus IFNγ-induced ICAM1 expression in AF cells was mediated by CCL2 secretion. 

### 2.2. JAK2 and FAK/ERK Pathways Regulate ICAM1 and CCL2 Expression

**Figure 2:**
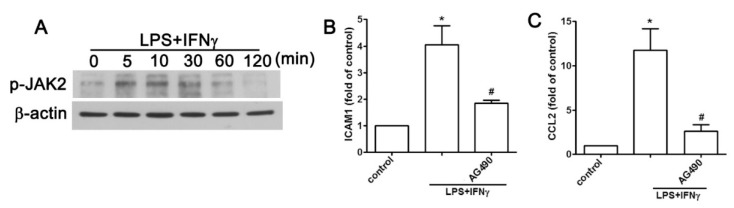
JAK2 pathway is involved in the pro-inflammatory stimuli-associated expression of ICAM1 and CCL2 in human AF cells. (**A**) Cells were treated with LPS plus IFNγ for 5, 10, 30, 60, or 120 min, and phosphorylated JAK2 expression was determined using western blot analysis. Cells were treated with the JAK2 inhibitor AG 490 (10 μM) for 30 min and with LPS plus IFNγ for another 6 h, and the expression of cell surface ICAM1 (**B**), and *CCL2* (**C**) were determined using flow cytometry and real-time PCR analysis, respectively. Quantitative data are presented as the mean ± SEM (representative of *n* = 3). * *p* < 0.05 compared with the control group. # *p* < 0.05 compared with the LPS plus IFNγ treatment group.

The JAK signaling cascade is a key pathway for immune responses and correlates with the expression of adhesion molecules in different cell types [21,22]. Previously, we demonstrated that LPS plus IFNγ enhances neuroinflammation through the activation of JAK2 [23]. Here we investigated whether JAK2 was involved in ICAM1 and CCL2 expression induced by LPS plus IFNγ. Increased phosphorylation of JAK2 with time was revealed when AF cells were exposed to LPS plus IFNγ (Figure 2A). In contrast, inhibition of JAK2 diminished the ICAM1 cell surface protein expression induced by LPS plus IFN (Figure 2B). Moreover, JAK2 inhibition resulted in decreased *CCL2* expression induced by LPS plus IFNγ (Figure 2C). These results indicated that CCL2 expression induced by LPS plus IFNγ was mediated by the JAK2 signaling pathway and resulted in ICAM1 expression in AF cells.

**Figure 3:**
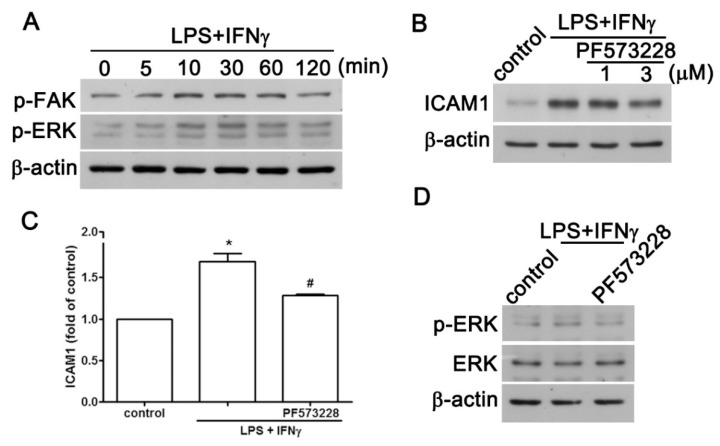
FAK/ERK is involved in the pro-inflammatory stimuli-associated expression of ICAM1 and CCL2 in human AF cells. (**A**) Cells were treated with LPS plus IFNγ for 5, 10, 30, 60, or 120 min, and expression of phosphorylated FAK1 and phosphorylated ERK were determined using western blot analysis. (**B**) Cells were treated with the FAK inhibitor PF573228 (1 or 3 μM) for 30 min and stimulated with LPS plus IFNγ for another 24 h. ICAM1 expression was determined using western blot analysis. Cells were treated with PF573228 (1 μM) for 30 min and treated with LPS plus IFNγ for another 6 h, and examined for cell surface ICAM1 (**C**) and CCL2 (**D**). Cells were treated with PF573228 (1 μM) for 30 min and stimulated with LPS plus IFNγ for another 60 min, and phosphorylated ERK expression was determined using western blot analysis. Quantitative data are presented as the mean ± SEM (representative of *n* = 3). * *p* < 0.05 compared with the control group. # *p* < 0.05 compared with the LPS/IFNγ treatment group.

Evidence has indicated that FAK is a critical regulator that contributes to ICAM1 modulation under various stimulations in different cell types [24,25]. Our previous report also showed that LPS plus IFNγ induces ERK activation in microglial cells [26,27]. To examine the role of FAK and ERK in LPS plus IFNγ-induced ICAM1 expression in AF cells, we conducted the following experiments to investigate the effects of FAK and ERK on ICAM1 expression. As shown in Figure 3A, LPS plus IFNγ increased both FAK and ERK phosphorylation in a time-dependent manner, being initiated at 10 min and sustained to 120 min. Inhibition of FAK by PF573228 dose-dependently diminished the effect of LPS plus IFNγ on total protein expression, and cell surface expression of ICAM1 (Figure 3B,C). Moreover, inhibition of FAK by PF573228 also reduced ERK phosphorylation (Figure 3D), suggesting that FAK is an upstream signal regulating ERK. 

### 2.3. GSK3 Signaling Pathway is Involved in the Enhancement of CCL2/ICAM1 

**Figure 4:**
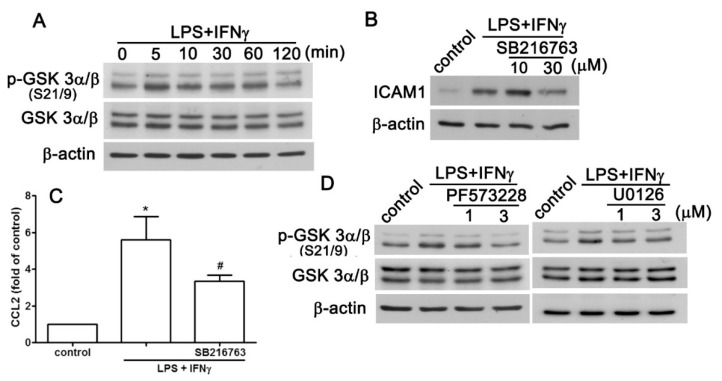
GSK3 is involved in the pro-inflammatory stimuli-associated expression of ICAM1 and CCL2 in human AF cells. (**A**) Cells were treated with LPS plus IFNγ for 5, 10, 30, 60, or 120 min, and phosphorylated GSK 3α/β expression were determined using western blot analysis. Cells were treated with the GSK3 inhibitor; SB216763, for 30 min and stimulated with LPS plus IFNγ for another 6 or 24 h. ICAM1 (**B**) and *CCL2* (**C**) expression were determined using western blot analysis and real-time PCR analysis, respectively. (**D**) Cells were treated with the FAK inhibitor PF573228 (left panel), or MEK inhibitor U0126 for 30 min and then treated with LPS plus IFNγ for another 60 min, and phosphorylated GSK 3α/β expression was determined using western blot analysis. Quantitative data are presented as the mean ± SEM (representative of *n* = 3). * *p* < 0.05 compared with the control group. # *p* < 0.05 compared with the LPS/ IFNγ treatment group.

GSK3 is involved in the modulatory effects in adhesion molecule expression, such as ICAM [28,29]. Here, we observed that LPS plus IFNγ induced GSK3 phosphorylation in a time-dependent manner in AF cells (Figure 4A). We also found that inhibiting GSK3 by SB216763 dose-dependently reduced ICAM1 protein expression induced by LPS plus IFNγ (Figure 4B). In contrast, the expression of CCL2 induced by LPS plus IFNγ was also diminished by inhibiting GSK3 (Figure 4C). We further investigated the regulatory mechanism of FAK and ERK on GSK3. As shown in Figure 4D (left panel), inhibition of FAK by PF573228 dose-dependently diminished GSK3 phosphorylation. In addition, inhibiting ERK by U0126 also abrogated GSK3 phosphorylation induced by LPS plus IFNγ (right panel, Figure 4D). These results suggested that GSK3 activation induced by LPS plus IFNγ is regulated by the FAK/ERK signaling pathway.

### 2.4. PKCδ is Involved in LPS Plus IFNγ-Induced Expression of CCL2/ICAM1 in AF Cells

**Figure 5:**
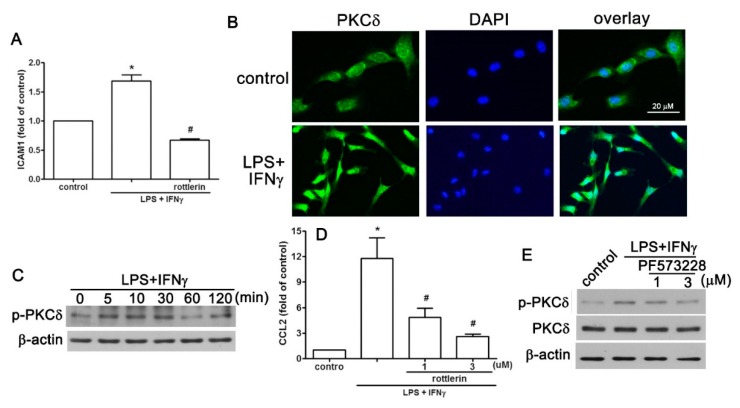
PKCδ translocation is involved in the pro-inflammatory stimuli-associated expression of ICAM1 and CCL2 in human AF cells. (**A**) Cells were treated with the PKCδ inhibitor rotterin for 30 min and stimulated with LPS plus IFNγ for another 24 h. Cell surface ICAM1 was determined using flow cytometry. (**B**) Immunofluorescence staining showed that LPS plus IFNγ stimulation resulted in PKCδ translocation. 4′,6-Diamidino-2-phenylindole (DAPI) was used as the nuclear counterstain. Scale = 20 μm. (**C**) Cells were treated with LPS plus IFNγ for 5, 10, 30, 60, or 120 min, and the expression of phosphorylated PKCδ expression was determined using western blot analysis. (**D**) Cells were treated with rotterin for 30 min and stimulated with LPS plus IFNγ for another 6 h, and *CCL2* expression was determined using real-time PCR analysis. (**E**) After treating with the FAK inhibitor PF573228, cells were stimulated with LPS plus IFNγ for another 60 min, and the expression of phosphorylated PKCδ was determined using western blot analysis. Quantitative data are presented as the mean ± SEM (representative of *n* = 3). * *p* < 0.05 compared with the control group. # *p* < 0.05 compared with the LPS plus IFNγ treatment group.

Early reports demonstrated that PKCδ is an important regulator in ICAM1 expression via the mediation of a variety of biological functions [30,31]. We observed that rottlerin-mediated inhibition of PKCδ by rottlerin significantly reduced the cell surface expression of ICAM1 induced by LPS plus IFNγ (Figure 5A). This prompted us to examine PKCδ activation regulated by LPS plus IFNγ. The level of phosphorylated PKCδ was significantly elevated in AF cells (Figure 5C). Immunofluorescence staining revealed the translocation of PCKδ translocation from the cytoplasm to the nucleus after stimulation of AF cells with LPS plus IFNγ (Figure 5B). Rottlerin treatment dose-dependently reduced *CCL2* enhancement induced by LPS plus IFNγ (Figure 5D). Inhibiting focal adhesion kinase (FAK) by PF573228 also dose-dependently reduced PKCδ phosphorylation induced by LPS and IFNγ (Figure 5E). These results suggest that PKCδ is a critical transcription factor of CCL2 and ICAM1 expression in AF cells.

## 3. Discussion

Degeneration of IVDs involves structural disruption and cell-mediated compositional changes, and is associated with low back pain [32,33,34,35]. A specific pharmacological treatment for degenerative disc disease is still needed [20]. IVD degeneration is associated with inflammation, which can become chronic [36]. Beyond the potential application to new therapeutics, the novel hypotheses and mechanistic studies described here have the potential to determine how the pathophysiologic state in IVD is modulated. Elevated inflammatory mediators and pro-inflammatory cytokines have been observed in degenerated and herniated IVD tissues [4,7]. In addition, herniated disc tissue displays increased infiltration of inflammatory cells with abundant levels of pathology regulatory cytokines, including tumor necrosis factor-alpha (TNF-α), interferon-gamma (IFNγ), and interleukin-1β [37]. We previously reported that the endotoxin component of LPS plus IFNγ causes an imbalance of inflammatory responses [38]. Patients with radicular pain caused by lumbar spinal stenosis who received epidural injections of a monoclonal antibody directed at the IL-6 receptor, displayed reductions in lower back and leg pain [39]. Moreover, epidural treatment of the spinal nerve with an inhibitor of the pro-inflammatory cytokine TNF was reported to provide pain relief in patients experiencing radicular pain arising as a result of lumbar spinal stenosis [40]. Treatment with a neutralizing antibody for TNF-α was shown to produce a marked improvement in leg pain and a reduction in the number of patients with disc herniation-associated sciatica [41,42]. 

Angiogenesis and macrophage infiltration around herniated tissue was observed in immunocytochemical study of disc herniation tissue [43,44]. Moreover, inflammatory cells were observed infiltrating the herniated tissue and in enhancement at the site of herniation [10]. In addition, monocytes/macrophage infiltrate the epidural tissue around the herniation that break down and digest the herniated tissue [45,46]. Several cytokines are involved in the development of inflammation and are responsible for the pathology of IVD degeneration [47]. Importantly, ICAM1 has also been found in herniated IVD tissues [14] and is promoted in inflammatory states by pro-inflammatory cytokines [37,48]. ICAM1 is involved in the binding of leukocytes and likely other immune cells, facilitating their infiltration into tissue [49]. Interestingly, IFNγ was demonstrated to be a major co-stimulant, along with pro-inflammatory cytokines, to enhance the expression of ICAM1 in IVD [49]. 

Several cytokines and chemokines are involved in the development of inflammation and are responsible for the pathology of IVD degeneration [47]. NP cells from degenerate human IVDs have been reported to be a source of CCL2 [50]. Expression of CCL2 and ICAM1 is strongly associated in endothelial cells [51]. Importantly, the recruitment of myeloid cells into the lumbar region occurs with concomitant increases in CCL2 and ICAM1 after spinal cord injury [52]. Activation of spinal perivascular macrophages is accompanied by the consistently increased expression of ICAM1 and the chemokine MCP-1 during experimental allergic encephalomyelitis [53]. Surprisingly, CCL2 has also been reported to negatively regulate ICAM1 expression and breast cancer metastasis [54]. Moreover, CCL2 induces the expression of ICAM1 on human lymphatic endothelial cells and facilitates the attachment of cancer cells to lymphatic endothelial cells [55]. As shown in Figure 1E, treatment of CCL2 neutralizing antibody partially antagonized the enhancement of ICAM1 expression. We cannot exclude the possibility that there may be other molecules involved in the regulation of ICAM1 expression in AF cells. In addition, this study was limited to in-vitro settings. The detailed mechanisms involved in IVD degeneration and disc herniation require further study.

GSK3α/β modulates numerous cellular functions through phosphorylation-mediated changes in its activation and inhibition [56]. GSK3 α/β is activated by phosphorylation of the Tyr279/216 residues, and inactivated by the phosphorylation of Ser21/9 residues [57,58]. GSK3 promotes production of the pro-inflammatory mediators IL-6, TNF-α, and IFNγ in monocytes and microglia [59,60]. In addition, GSK3α/β has also been studied concerning the production of IL-8 in mononuclear phagocytes infected with *Burkholderia cenocepacia* [61]. Interestingly, modulation of GSK3α/β activity by Tyr279/216 phosphorylations negates ICAM1 expression [62]. In addition, the GSK3 inhibitor CHIR99021 reduces ICAM-1 expression in adipose tissue and skeletal muscle of gestational diabetes [63]. We previously reported that phosphorylation of GSK3α/β at Ser 21/9 residues regulates an alternative type of inflammation in microglial cells [64]. PKCδ is a novel serine/threonine kinase of the PKC family that is involved in cell proliferation, differentiation, migration, survival, and apoptosis [65]. PKCδ has also been reported to participate in anti-inflammatory activities and neuroprotection [66,67]. We reported that PKCδ modulates suppressors of cytokine signaling-3 expression and anti-inflammatory effects in microglia [68]. PKCδ-knockout mice were markedly resistant to disc degeneration in an experimental disc injury model [69]. In addition, PKCδ suppresses the transcriptional activity of c-fos in NP cells [70]. 

## 4. Materials and Methods

### 4.1. Reagents

Recombinant murine IFNγ was purchased from PeproTech (Rocky Hill, NJ, USA). LPS from *Escherichia coli* serotype 055:B5 was obtained from Sigma-Aldrich (St. Louis, MO, USA). Dulbecco’s modified Eagle medium, and fetal bovine serum (FBS) were purchased from Gibco-BRL (Invitrogen Life Technologies, Carlsbad, CA, USA). Secondary antibodies, primary antibodies against phospho-ERK1/2, ERK2, FAK, and β-actin were purchased from Santa Cruz Biotechnology (Santa Cruz, CA, USA). Primary antibodies against phospho-FAK, phospho-GSK3α/β, GSK3α/β, and PKCδ (phosphorylated at Thr^505^) were purchased from Cell Signaling Technology and Neuroscience (Danvers, MA, USA). Pre-immune rabbit IgG was purchased from Abcam (Cambridge, MA, USA). LY294002, PP2, SB216763, PPACK, and diaminobenzene were also obtained from Sigma-Aldrich. U0126 was obtained from Tocris Bioscience (Ellisville, MO, USA). AG490 and PF573228 were purchased from Calbiochem (La Jolla, CA, USA). The avidin-biotin complex standard kit was purchased from Vector Laboratories (Burlingame, CA, USA).

### 4.2. Human AF Cells

Healthy human AF cells from the human IVD were purchased from ScienCell Research Laboratories (Cat# 4810; Carlsbad, CA, USA). Cells were cultured in NP cell medium with supplements (Cat. #4801; ScienCell Research Laboratories) on poly-L-lysine-coated tissue culture dishes. The medium was changed every 2 days, and cells were passaged when they were over 90% confluent. Cells were cultured in a medium supplemented with 10% heat-inactivated FBS, 100 U/mL penicillin, and 100 mg/mL streptomycin at 37 °C, and incubated in a humidified atmosphere consisting of 5% CO_2_ and 95% air. The cell cultures from passage 5–10 were used for the experiments to analyze the mechanism of pro-inflammatory stimuli modulating the expression of the ICAM1 adhesion molecule.

### 4.3. Western Blot Analysis

The western blotting procedure has been previously described [71]. Briefly, cells were lysed with immunoprecipitation assay buffer on ice. Protein samples were separated by sodium dodecyl sulfate-polyacrylamide gel electrophoresis, and transferred to polyvinylidene difluoride membranes. The membranes were blocked with 5% nonfat milk and then probed with the appropriate primary antibody. The blots were visualized using enhanced chemiluminescence using LAS-3000 film. The blots were subsequently stripped by incubation in a stripping buffer and re-probed for β-actin as a loading control. Quantitative data were obtained using a densitometer and ImageJ software.

### 4.4. RNA Extraction and Quantitative Real-Time PCR

Total RNA was extracted from cells using a TRIzol kit (MDBio Inc., Taipei, Taiwan). The reverse transcription reaction was performed using 2 μg of total RNA that was reverse transcribed into cDNA. Quantitative real-time PCR was performed using SYBR-green detection of PCR products in real-time using the 96-well StepOne Plus Real-Time System (Applied Biosystems, Foster City, CA, USA). Primers used for quantitative PCR were as follows: *ICAM1*: 5′-CCCCCCGGTATGAGATTGT-3′ and 5′-GCCTGCAGTGCCCATTATG-3′; *CCL2*: 5′-AAGGGCTCGCTCAGCCAGATGC-3′ and 5′-GGAATCCTGAACCCACTTCTGC-3′; and *GAPDH*: 5′-AGGGCTGCTTTTAACTCTGGT-3′ and 5′-CCCCACTTGATTTTGGAGGGA-3′.

### 4.5. Immunocytofluorescence Staining

Cells were seeded onto glass coverslips and exposed to pro-inflammatory stimuli for 1 h, fixed with 4% paraformaldehyde for 15 min, and then permeabilized with Triton X-100 for another 30 min. Cells were incubated with anti-PKCδ antibody for 1 h at room temperature. After a brief wash to remove the antibody, cells were incubated with an Alexa 488 efluor-conjugated secondary antibody (Invitrogen Life Technologies). Finally, cells were mounted and visualized with a digital fluorescence microscope.

### 4.6. Statistical Analysis

Statistical analysis was performed using the GraphPad Prism 6 (GraphPad Software Inc., San Diego, CA, USA). The values are presented as the mean ± SEM Statistical difference between the experimental group and the control group was assessed using Student’s *t*-test. 

## 5. Conclusions 

The present results reveal that FAK/ERK/GSK3α/β and PKCδ are important signaling pathways in the inflammatory responses in AF. Targeting the CCL2/ICAM1 pathways may be pivotal in the preventive treatment of inflammatory responses that aggravate the process of disc degeneration. The present findings may contribute to the diagnosis and development of post-operative strategies for IVD degeneration and disc herniation.
